# Addition of dexmedetomidine and neostigmine to 1.5 % lidocaine and triamcinolone for epidural block to reduce the duration of analgesia in patients suffering from chronic low back pain

**DOI:** 10.25122/jml-2019-0043

**Published:** 2019

**Authors:** Shima Zargar, Ali Nazemi Rafie, Alireza Sosanabadi, Alireza Kamali

**Affiliations:** 1. Department of Anesthesiology and Critical Care, Arak University of Medical Sciences, Arak, Iran; 2. Department of Neurosurgery, Arak University of Medical Sciences, Arak, Iran

**Keywords:** Lower back pain, Epidural injection, Neostigmine, dexmedetomidine hydrochloride

## Abstract

Lower back pain is one of the leading causes of disability in the world. The aim of this study was to evaluate the effect of supplementation of dexmedetomidine and neostigmine with lidocaine 1.5% and triamcinolone for epidural block in increasing the duration of analgesia among patients suffering from chronic low back pain. In this double-blind, randomized clinical trial, 33 patients with chronic low back pain were included in three groups of 11 patients for epidural blockage. Triamcinolone (40 mg/ml) was added to lidocaine 1.5% solution (2 cc/segment) for all three groups. In group N, neostigmine was used at a dose of 1 mg (mg), followed by group D (dexmedetomidine 35 μg [0.5 μg/kg]), and grou [ND (neostigmine 0.5 mg, and 35 μg dexmedetomidine, all of which were added to the triamcinolone and lidocaine solution in each group. Medications were injected into the epidural space using an interlaminar approach. Subsequently, scores of pain and duration of analgesia were recorded in questionnaires and analysed using SPSS version 23. One month after the injections, pain scores recorded in the N group were 7.6±1.4, followed by 5.88±1.2 in group D and 5.42 ±1.1 in group ND. Therefore, the pain scores were significantly higher in the neostigmine group than the other two groups (p = 0.02), but no significant difference was found between the two groups that received dexmedetomidine and a combination of dexmedetomidine + neostigmine. Three months after the injections, there was a significant difference in pain scores between the two groups (P = 0.01). Both neostigmine and dexmedetomidine were capable of reducing the pain of patients with chronic low back pain after epidural block. However, neostigmine’s impact is lower compared to dexmedetomidine. The combination of the two drugs also reduced the pain scores of the patients after the intervention.

## Introduction

Over the past two decades, extensive epidemiological studies have been carried out on low back pain and indicate high prevalence and high direct and indirect costs in areas such as medical, legal, employment (absenteeism), products, and inadequate quality of life [1, 2]. More than 80% of the population will experience at least one episode of low back pain during their life [2, 3].

According to estimates, approximately 1% to 2% of the active population in the United States suffers from severe disability due to back pain, and 12% to 15% of annual visits of patients in American healthcare centers have been attributed to back pain. 12% to 15% of annual visits to patients in American health care centers due to low back pain. In Iran, back pain is one of the issues that is common in the society, with high prevalence among high school students (17%), nurses (62%) and pregnant women (84%) [1]. Most patients suffering from acute low back pain usually experience symptoms of healing thanks to preservative or spontaneous therapies within 4 to 6 weeks. In the event of symptoms persisting for more than three months, we talk about chronic low back pain [3, 4]. The main goal in the treatment of chronic low back pain is to relieve pain temporarily so that the patient can participate in therapeutic exercises and a coherent rehab program.

In this way, the strength and mechanics of the patient’s body improve, physical stress will be minimized, and pain will be resolved for a longer period [4] Among the less invasive methods, epidural injections are one of the most commonly used measures to reduce low back pain and lower limb pain, leading to improvement of the quality of life [5]. An epidural injection is especially beneficial in patients whose chronic pain does not respond to conservative and physiotherapy treatments [6].

Despite extensive research in this area, there is still controversy over the number of epidural infusions and their maximum frequency, and the administration route of the epidural injection (transforaminal, interlaminar and caudal). In addition, the researchers still failed to reach agreement on the ideal volume of consumed drugs, injections or non-injections of corticosteroids, drug alternatives as to the main epidural anesthetic drug, adjuvant drugs and the effectiveness of these methods in reducing patient’s pain [5-12]. Different adjuvants have been used in combination with local anesthetics to improve analgesia and to reduce complications, but none have been accepted [13]. Meanwhile, dexmedetomidine and neostigmine are effective adjuvants used in the lumbar epidural blocks [14, 15]. Neostigmine is an acetylcholinesterase inhibitor, and its analgesic effects are owed to increased acetylcholine concentrations in the posterior spinal cord and meninges. According to some studies, the use of neostigmine in combination with local anesthetics and opioids has a synergistic and postoperative effect, which increases the duration of analgesia and decreases the VAS (visual analogue scale) score [16].

Other adjuvant drugs include dexmedetomidine, which is a highly sensitive and highly selective α-2 adrenergic receptor agonist with a tendency of more than 8-fold in comparison with clonidine, thus reducing undesired side effects of alpha-1 receptors [12, 17]. Various studies have shown that dexmedetomidine is capable of increasing the sensory block, motor block and also the duration of analgesia [17]. According to these interpretations, this study was aimed to compare the effect of two drugs (neostigmine and dexmedetomidine) as adjuvant drugs along with lidocaine and corticosteroids in increasing the duration of analgesia in patients suffering from chronic low back pain for improving the quality of life among these patients.

## Materials and Methods

This study was a double-blind, randomized clinical trial on all patients with chronic low back pain who were considered an epidural block candidate at the Amiralmomenin Hospital and Vali Asr Hospital in Arak, Iran.

### Inclusion criteria

1. All patients with chronic low back pain who were candidates for the lumbar epidural block. 2. All patients with the intent to participate in the study. 3. Patients aged 30-70 years 4. Patients with physical status ASA I or II. 5. Absence of vitamin D deficiency.

### Exclusion criteria

1. Patients outside the age range of 30-70 years 2. Patients who were not candidates for an epidural block. 3. Patients with hypersensitivity to lidocaine neostigmine and dexmedetomidine. 4. Patients with physical status ASA greater than II (III, IV). 5. Patients with failed epidural blocks. 6. Female patients who are pregnant or lactating. 7. Patients with a history of fracture. 8. Patients who are suffering from uncontrolled psychological disorders.

In this study, all patients with chronic low back pain (back pain for more than 6 weeks), candidates for epidural block with physical status ASA I or II, who have never had an allergic reaction to local anesthetics and epidural anesthesia were randomly divided into three groups: N (Neostigmine), D (Dexmedetomidine), and ND (Neostigmine and Dexmedetomidine). Patients who met the inclusion criteria and provided written informed consent were included in the study. At first, the Ringer solution of 5 cc/kg was injected 10 to 15 minutes before the epidural block for adequate hydration for each patient of the three groups. Afterward, the patients were placed in a sitting position, and the subjects received epidural blocks at the L4-L5 or L5-S1 level using the interlaminar approach using a 19- or 20-gauge epidural catheter. After entering the epidural space and confirming the correctness of needle placement, the drug combination was injected. In all three groups, 2 cc/segment of triamcinolone (40 mg/ml) was added to the lidocaine 1.5% solution, followed by injection.

In group N, neostigmine was used at a dose of 1 mg (mg), followed by group D (dexmedetomidine 35 μg [0.5 μg/kg]) and group ND (neostigmine 0.5 mg, and 35 μg dexmedetomidine, all of which were added to the triamcinolone and lidocaine solution in each group. The volume of the injectable solution was mixed with distilled water up to 20 cc for each of the three groups.

During epidural blocking, SPO2 (pulse oxygen saturation), PR (pulse rate) and NIBP (non-invasive blood pressure) were monitored. Ultimately, after performing the procedure and ensuring that the hemodynamic status of the patient was stable, they were transferred to the recovery room. Complete monitoring (including SPO2, PR, NIBP,) was continued even after the patients were transferred to the recovery room.

### Double-blind procedure

This double-blind study included patients who provided written informed consent, the injectable solution composition being unknown. Furthermore, the healthcare professional responsible for completing the questionnaires and recording the pain and disability scores, was not aware of the patients in the studied groups and did not know about the epidural block, only completing questionnaires based on the numbers assigned in the operating room by an anesthesiologist responsible for the study (who performed the procedure).

A total of 33 patients with chronic low back pain who met the inclusion criteria were randomly assigned to three groups (N, D, and ND) using a randomized table. Therefore, 11 patients were assigned to each group.

### Data collection

The pain score and duration of analgesia were evaluated based on the visual analogue scale (VAS) in the recovery room, one month and three months after the procedure. Improvement of performance was also evaluated based on the score of disability (ODI - Oswestry Disability Index) before and one month after the intervention. The duration of analgesia was recorded on the basis of a painkiller request (in terms of days); therefore, all patients were requested to return to the clinic or address to a pain management clinic if the pain would return. The exact duration of analgesia was recorded in the questionnaires to track the patients’ pain. Also, other patient information, including demographic data, hemodynamic status (SPO2, PR, NIBP) during the procedure and recovery, as well as the mean time needed for movement onset were recorded by questionnaires.

#### Sample size and sampling method

According to the type of study (randomized clinical trial), 33 patients who met the inclusion criteria and provided written informed consent, were randomly assigned to three equal groups: N (neostigmine), D (dexmedetomidine) and DN (dexmedetomidine and neostigmine).

**Figure:**
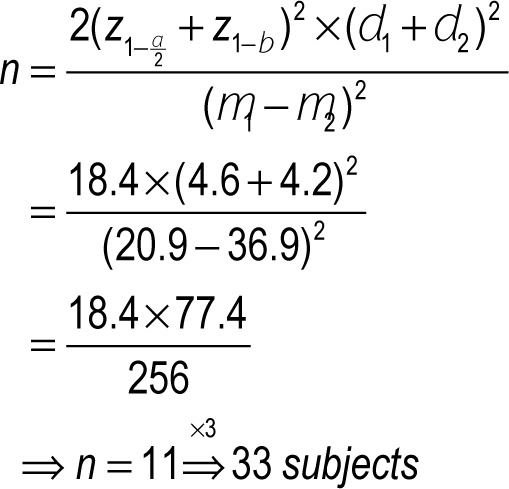


**Figure:**
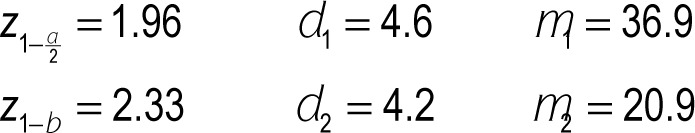


### Data analysis

In this study, SPSS 19 software was used to analyze questionnaires data. Statistical analysis was performed using T-test and ANOVA. Finally, the results were shown in Tables and graphs.

### Ethical considerations

In this study, the names and characteristics of the subjects were confidential and costs were not imposed on the patient’s family and hospital. In addition, completion of the form and the patients were satisfied after receiving training providing written consent. At all stages of the research, such as drafting the proposal, collecting samples and analyzing data, the researchers were required to respect the ethical provisions issued by the Ministry of Health and the Helsinki Declaration. This research was approved by the Ethics Committee of the Arak Medical University Research Council (IR.ARAKMU.REC.1395.256).

## Results

A total of 11 patients were enrolled in each group; the mean age and distribution of the sexual abnormalities of these patients are listed in Table 1. According to Table 1, there was no significant difference between the three groups in terms of mean age since the average age of the patients was similar (P = 0.3). Regarding the distribution of sexual frequency, there was no significant difference either (the frequency of male subjects was similar in the three groups - 55%; P = 0.4).

**Table 1:** Comparison of mean age and sex distribution of patients with chronic low back pain of the three groups

Mean age/Group	Neostigmine group		Dexmedetomidine group	Combined group	P-value
Age average	41/7+/-2/3		42/4-/+ 1/9	43/3 -/+ 2/7	P= 0.3
					No significance
Frequency distribution	Male	57.1%	55.4%	56.6%	P= 0.4
	Female	42.9%	44.6%	43.4%	No significance

Based on Table 2, no significant difference was found between the three groups regarding the duration of chronic low back pain before the intervention; the duration of back pain was found to be similar in all three groups, which was almost equal to 1.7 years (p = 0.6).

**Table 2:** Comparison of mean back pain duration before intervention in patients with chronic low back pain of the three groups

Average back pain duration /group	Neostigmine group	Dexmedetomidine group	Combined group	P-value
Average back pain duration (Year)	1/8+/-0/85	1/65+/-0/56	1/78+/-0/68	P= 0.6
				No significance

As indicated in Table 3, no significant difference was observed between the three groups in terms of recovery pain scores and almost every score was revealed to be less than 0.5. However, pain scores were different in the three groups one month after the injection, the pain scores in the neostigmine group being significantly higher than the other two groups (p = 0.02), but no significant difference was observed between the neostigmine group and dexmedetomidine group compared to the combined group (dexmedetomidine+ neostigmine).

**Table 3:** Comparison of patients’ pain scores at different times after the intervention in patients with chronic low back pain of the three groups.

Average pain/group	Neostigmine group	Dexmedetomidine group	Combined group	P-value
Pain score in the recovery room	0/38+/-0/22	0/25+/-0/18	0/21+/-0/16	P= 0.4
				No significant
Pain score 1 month after the intervention	7/9+/-6/7	5/88+/-1/2	5/42+/-1/1	P= 0.02
				Significant
Pain score 3 months after the intervention	7/1+/-1/1	4/1+/-0/85	4/1+/-0/64	P= 0.01
				Significant

Nevertheless, pain scores were significantly different at three months after the injection; the pain scores in the neostigmine group were higher than the other two groups (p = 0.01), but no significant difference was revealed between the dexmedetomidine and combined group (dexmedetomidine + neostigmine).

As Table 4 suggests, the duration of analgesia in the neostigmine group was significantly lower as compared to the other two groups (P = 0.03), while there was no significant difference between the dexmedetomidine and combined group. In other words, the effect of dexmedetomidine was far more pronounced than that of neostigmine, either alone or in combination.

**Table 4:** Comparison of the duration of post-injection analgesia in patients suffering from chronic low back pain of the three groups.

Analgesia/group	Neostigmine group	Dexmedetomidine group	Combined group	P-value
Duration of analgesia (days)	14/8+/-2/9	16/1+/-2/3	16/7+/-2/7	P=0.03
				Significant

According to Table 5, no significant difference between was observed between the three groups regarding movement after the intervention, and the same result was observed in all three groups (2.5 hours; p = 0.06).

**Table 5:** Comparison of the mean time needed for movement onset after the intervention in patients with chronic low back pain of the three groups.

Mean time needed for movement onset/groups	Neostigmine group	Dexmedetomidine group	Combined group	P-value
Movement onset (In hours)	2/87+/-0/65	2/61+/-0/98	2/4+/-0/66	P=0.6
				No significance

Our findings showed no significant difference between the three groups in terms of ODI scoring before the intervention; almost all ODI scores were the same in all three groups (45%; p = 0.06). However, the ODI score was significantly higher in the neostigmine group after the intervention when compared with the other two groups (P = 0.02), but no significant difference was found between the two groups and the combined group (Table 6).

**Table 6:** Comparison of the ODI Score before and after the intervention in patients with chronic low back pain of the three groups.

Group/score ODI	Neostigmine group	Dexmedetomidine group	Combined group	P-value
ODI Score before intervention	45.8%	46.4%	47.1%	P= 0.6
				No significant
ODI Score after intervention	42.4%	38.7%	38.1%	P= 0.02
				Significant

As shown in Table 7, no significant difference was determined between the three groups in terms of mean blood pressure, heart rate and arterial oxygen saturation (p=0.6, p = 0.4). Approximately, all groups showed similar hemodynamic parameters in the recovery room.

**Table 7:** Comparison of mean blood pressure, heart rate and arterial oxygen saturation in patients suffering from chronic low back pain in the recovery of the three groups.

Mean/group	Neostigmine group	Dexmedetomidine group	Combined group	**P-value**
**MAP mean**	88/2+/-1/1	87/6+/-3/4	86/6+/-2/7	P= 0.4
				No significance
**PR mean**	82/6+/-3/1	81/8+/-3/7	82/2+/-2/9	P= 0.6
				No significance
**mean SPO2**	96.6%	97.4%	96.2%	P= 0.6
				No significance

## Discussion

Achieving an appropriate combination to increase the duration of analgesia in patients with chronic low back pain is one of the specific goals of anesthetics and pain management specialists. In our study, the comparison of the addition of dexmedetomidine and neostigmine to lidocaine 1.5% and triamcinolone for epidural block in patients suffering from chronic low back pain were studied. The results indicated that pain scores of patients of all three groups in the recovery room had dropped dramatically, but did not show any significant difference. However, pain scores were significantly different at one and three months after the intervention. Specifically, the pain scores in the dexmedetomidine group (D) and the combined group (D + N) were significantly lower than the neostigmine (N) group, but there were no differences between the dexmedetomidine and combined group).

The mean time needed for movement onset of patients was the same for all three groups, and analgesia duration was not significantly different among the three groups within 24 hours after the intervention. Comparison of the ODI scores in the three groups revealed that the scores were significantly reduced at one-month after the intervention in the dexmedetomidine and combined group compared to the neostigmine group. However, there was no significant difference between the dexmedetomidine and the combined group.

Our results are consistent with the previous studies. Nourmohammadpour et al. conducted a study in 2016 on 7889 Iranian patients aged 30-70 years with neck pain, back pain, and chronic knee pain. The prevalence rates of chronic neck pain, chronic low back pain, and chronic knee pain were 15.3%, 27.18 % and 29.97%, respectively. In the aforementioned study, in addition to identifying the risk factors for chronic low back pain and neck pain, the use of regional blocks has been recommended to reduce chronic pain in cases of chronic low back pain and neck pain. In our study, epidural block was capable of significantly reducing the pain of patients with chronic low back pain, which was consistent with the results of the above-mentioned study [19].

In another study conducted by Ackerma et al. in 2007, 90 patients aged 18-60 years with L5-S1 disc and chronic low back pain were evaluated. It has been determined that epidural injection using a caudal, intraluminal, and trans-foraminal approach have been effective in the chronic pain of the patients included in the study. Other results of the study mentioned above indicated that trans-foraminal epidural steroid injections were more effective compared to the caudal and intraluminal approach [6], results which were consistent with our study results as well because we also found that steroid injection by epidural block method leads to the reduction of pain in patients with chronic low back pain.

In another study, the effect of lumbar epidural block on patients with chronic low back pain has been investigated, where patients were divided into two groups: local anesthetic alone and local anesthetic in combination with betamethasone. The second group (combined group) showed a significant reduction in the ODI and pain scores compared to the first group (local anesthetic alone). The ODI score was found to be significantly reduced in 69% of patients of the first group and 83% of patients of the second group. Therefore, epidural injections of steroids together with local anesthetics have been shown to be effective in reducing chronic back pain [19].

These results were consistent with our findings; although all of the three local anesthetics and steroid anesthetics were administered epidurally in our study, the ODI and pain scores were reduced in our patients.

In 2015, Chou et al. discussed pain management injection therapies for patients suffering from low back pain, where epidural steroid injections for preventing the pain caused by chronic radiculopathies led to an immediate improvement in the function and reduced the pain of the patients.

However, the benefits of these injections were short and unstable and did not affect the need for long-term surgical care [20]. The epidural steroid injection, along with the local anesthetic, resulted in decreased pain and ODI scores (the result was consistent with ours), but a significant number of patients showed decreased pain scores three-months after the intervention, different from what Chou et al. stated.

Comparison of our study with prior research reports showed that our results were consistent with most of the previous studies. In our study, as in most previous studies, the injection of local and steroid anesthetics has led to a reduction in the chronic pain of the patients. Moreover, the addition of two adjuvants (neostigmine and dexmedetomidine) has led to a reduction in the pain and ODI scores. Regarding the use of both dexmedetomidine and neostigmine in the present study and their appropriate effects as an adjuvant along with local anesthetics and steroids, the final effect of these two adjuvants was compared to eventually provide an appropriate combination for increasing the duration of analgesia.

## Conclusions

After epidural block, both neostigmine and dexmedetomidine, along with local anesthetics and steroids, were capable of reducing the pain of patients suffering from chronic low back pain. Neostigmine has a lower effect on pain when compared to dexmedetomidine. Furthermore, the combination of the two drugs also reduced the pain scores of the patients after the intervention, although this reduction was not significant in comparison with the dexmedetomidine group.

## Acknowledgments

We are grateful to the Clinical Research Center of Valiasr Hospital, the operative’s operating room for Valiasr Hospital and the surgical team of Amir Al-Momenin Hospital.

## Conflict of Interest

The authors confirm that there are no conflicts of interest.
